# Asymptomatic peripheral vascular disease in total knee arthroplasty: preoperative prevalence and risk factors

**DOI:** 10.1007/s10195-014-0305-z

**Published:** 2014-07-22

**Authors:** Ill Ho Park, Su Chan Lee, Il Seok Park, Chang Hyun Nam, Hye Sun Ahn, Ha Young Park, Viralkumar Harilal Gondalia, Kwang Am Jung

**Affiliations:** Joint and Arthritis Research, Department of Orthopaedic Surgery, Himchan Hospital, 20-8, Songpa-dong, Songpa-gu, Seoul, Korea

**Keywords:** Vascular disease, TKA, DVT, Arterial complications

## Abstract

**Background:**

Although vascular disease is commonly accepted as a risk factor for wound complications and prosthetic joint infections, little is known about the preoperative prevalence of lower-extremity peripheral vascular disease in patients undergoing total knee arthroplasty (TKA). In this study, we investigated the prevalence of asymptomatic vascular disease and its risk factors.

**Materials and methods:**

A total of 1,000 knees of 692 patients who underwent primary TKA due to osteoarthritis were preoperatively evaluated by experienced musculoskeletal radiologists using Doppler ultrasonography of the lower extremity vessels. The mean age of the patients was 74.1 years (range 65–81). Risk factors for development of peripheral vascular disease were investigated.

**Results:**

Abnormal findings were identified in 38 knees of 32 patients (4.6 %); atherosclerotic changes in 31 knees of 25 patients (3.6 %), deep vein thrombosis (DVT) in two knees, and anomalous vessels in five knees. Three out of 31 knees with atherosclerotic changes showed severe luminal stenosis. Two knees were moderate and 26 knees showed mild changes according to our institutional criteria. Multivariate logistic regression analysis showed that age and diabetes mellitus were positively associated with vascular pathology.

**Conclusion:**

The prevalence of incidentally detected peripheral vascular disease was significant. Three of 31 knees had severe arterial stenosis and two knees had DVT. All patients with vascular pathologies had one or more risk factors related to vascular disease. Out of those patients, age was the most important risk factor. Understanding the prevalence of vascular pathology and related risk factors in TKA candidates may be important for successful TKA.

**Level of Evidence:**

Level III.

## Introduction

Vascular complications after total knee arthroplasty (TKA) are rare but can be limb-threatening and sometimes life-threatening events. The incidence of arterial complications ranges from 0.03 % to 0.17 % [1, 2]. Limb-threatening complications include poor wound healing, deep infection and so on. The amputation rate is reported to be 25–43 % in patients with arterial complications after TKA [3, 4]. Although underlying vascular disease is commonly accepted as an identifiable risk factor for arterial insufficiency that can lead to wound complications and periprosthetic joint infection, little is known about the preoperative prevalence of lower extremity peripheral vascular disease in patients undergoing TKA. In this study, we investigated the prevalence of asymptomatic vascular disease and its risk factors in TKA candidates.

## Materials and methods

Between January and May 2010, a total of 1,000 knees of 692 patients scheduled for primary TKA for the treatment of osteoarthritis were preoperatively evaluated with Doppler sonography of the lower extremity vessels. The patients who had vascular-related symptoms, including intermittent pain, rest pain, or skin ulcers and had a previous history of percutaneous transluminal angioplasty (PTA) or bypass surgery, were excluded from this study. Doppler sonography was performed by experienced musculoskeletal radiologists with approximately ten years of clinical experience. The severity of peripheral vascular disease was graded as follows: mild—atherosclerotic plaque is present without luminal stenosis; moderate—luminal stenosis is present and multi-detector computed tomography (MDCT) is required; and severe—significant luminal stenosis identified with MDCT.

Risk factors for peripheral vascular disease were investigated in both groups. We reviewed demographic data including age, sex and body mass index (BMI). In addition, known risk factors including hypertension, diabetes mellitus, smoking, hyperlipidemia, and decreased renal function were investigated through the patients’ medical records. Multivariate logistic regression analysis was performed to analyze the risk factors of preexisting vascular disease and statistical analyses of respective parameters were conducted using the SPSS (18.0 for Windows, Chicago, IL, USA) statistical software program.

## Results

**Fig. 1:**
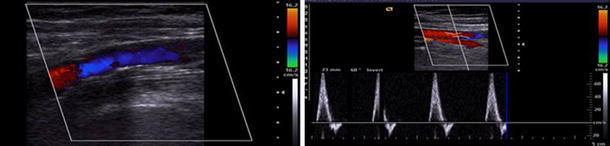
Doppler sonography. Severe luminal stenosis associated with diffuse atherosclerotic and calcified plaque

**Fig. 2:**
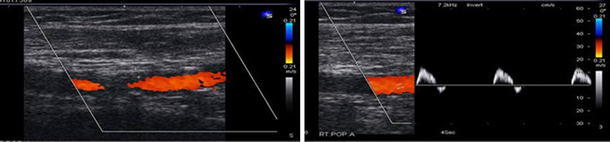
Doppler sonography. Mild diffuse atherosclerotic plaque changes without significant stenosis, but with decreased peak flow velocity on spectral mode

The mean age of the patients was 74.1 years (range 65–81), and the number of men and women were 62 and 630, respectively. The mean BMI of the patients was 25.4 kg/m^2^ (Table 1). Abnormal Doppler sonography findings were identified in 38 knees of 32 patients (4.6 %): atherosclerotic changes in 31 knees of 25 patients (3.6 %), deep vein thrombosis (DVT) in two knees, and anomalous vessels in five knees. Six of the patients with atherosclerotic changes had bilateral involvement. Three out of 31 knees with atherosclerotic changes showed severe luminal stenosis associated with diffuse atherosclerotic and calcified plaque on Doppler sonography, as well as abnormal waveform patterns on the spectral mode (Fig. 1). Two knees were moderate, and showed mild diffuse atherosclerotic plaque changes without significant stenosis, but with decreased peak flow velocity on spectral mode (Fig. 2). Twenty-six knees were mild according to our institutional criteria, and showed only multiple calcified atherosclerotic plaques without luminal stenosis and decreased peak flow velocity.

**Table 1:** Analysis demographic and baseline data

	Patient (knee)
Total number of patients	692 (1,000 knees)
Male:female	62:630
Mean age	74.1
Mean BMI (kg/m^2^)	25.4
Doppler sonography	
Normal	660 (962 knees)
Abnormal	32 (38 knees) (4.6 %)
Atherosclerosis	25 (31 knees) (3.6 %)
Deep vein thrombosis	2 (2 knees) (0.3 %)
Anomaly of vessels	5 (5 knees) (0.7 %)

The most commonly affected vessels were the popliteal artery (64.5 %) and the common femoral artery (61.3 %). Anomalous vessels in five knees represented high origins of the anterior tibial artery.

Pertinent medical backgrounds in this study included diabetes (DM) (51 % of patients with peripheral vascular disease), hypertension (HTN) (87 %), hyperlipidemia (74 %), abnormal renal function (19 %), and smoking (0 %).

Multivariate logistic regression analysis showed that age (OR 5.26, 95 % CI 2.23–12.43) and diabetes mellitus (1.25, 95 % CI 1.15–1.37) were positively associated with vascular pathology. Two knees (one patient) out of 38 knees of incidence of DVT were septic arthritis occurring after primary TKA, and they had a both revision.

## Discussion

Peripheral vascular disease is defined as decreased arterial perfusion to the lower extremities by any cause, and can be clinically identified by intermittent claudication or the absence of arterial pulses in the lower extremities. Although patients with subclinical status may be asymptomatic, the condition can become clinically significant through the worsening of thromboembolic lesions by a natural course or surgical events such as TKA. In particular, arterial complications following TKA can lead to delayed wound healing or skin necrosis, deep infections, and amputations of affected limbs. The development of septic conditions from periprosthetic joint infection in elderly patients can sometimes be life-threatening.

Many risk factors predisposing patients to arterial complications after TKA have been identified, including a history of arterial insufficiency, previous vascular surgery, absent or asymmetrical pedal pulses, preexisting atherosclerotic disease, presence of a popliteal aneurysm, and radiographic evidence of calcification of the distal superficial femoral artery or popliteal arteries [3–7].

In our study, the prevalence of peripheral vascular disease incidentally detected by Doppler sonography was 4.6 % (38 knees of 32 patients). Of these 38 knees, 31 knees showed arterial pathologies including atherosclerotic changes on Doppler sonography. Anomalous vessels (considered a normal variation) were present in five knees, and preoperative DVT was present in two knees. DeLaurentis et al. [5] previously demonstrated that only 24 (2 %) of 1,182 patients who underwent TKA in their series had an underlying peripheral vascular disease. The prevalence of peripheral vascular disease in population-based and clinical studies varies (5.1–38.9 % in diabetic patients, 2.6–12.2 % in nondiabetic patients) [8–11]. However, these previous studies uniformly concluded that the prevalence of vascular disease was higher in diabetic than nondiabetic patients, and that the incidence in patients with abnormal peripheral arterial findings increased with age [8–12].

Age, sex, diabetes, hyperlipidemia, hypertension, and smoking are significant risk factors for peripheral vascular disease of the lower extremities [15–17]. In our study, all of the patients with vascular pathologies had one or more of the risk factors mentioned above. Of these, age was the most important risk factor, followed by diabetes mellitus. Associated risk factors of not only peripheral vascular disease but also of sequent arterial complications should be assessed preoperatively to determine whether preoperative vascular consultation or immediate surgical revascularization is necessary. When a screening test such as Doppler sonography is highly suggestive of arterial insufficiency, the ankle-brachial index (ABI) should be determined. Because ABI represents the severity of ischemia, it is a useful method for the preoperative assessment of vascular patency. Proper consultation and interventions should be performed by vascular surgeons according to ABI values [1, 18].

In addition, the use of tourniquets and subsequent pressure on preexisting atheromatous plaques are associated with arterial ischemic complications after TKA [6, 13, 14]. The use of tourniquets during TKA is controversial. Most arterial complications after TKA are associated with tourniquet use, especially in previously pathological arteries [2, 4, 6, 19]. As a result, some authors have recommended that TKA should be performed without tourniquets in patients who are at risk [5, 6]. In addition, two prospective randomized studies suggested that TKA performed without tourniquets is safe [16, 18].

Orthopedic surgeons should understand mechanisms responsible for arterial complications after TKA and risk factors associated with arterial ischemia including preexisting vascular pathology. Careful preoperative assessment should be performed, particularly in elderly or diabetic patients, who are at especially high risk. If necessary, vascular consultations should be performed before TKA, and the use of tourniquets during TKA should be carefully considered to prevent arterial complications [20]. Although the preoperative prevalence of DVT is very rare, orthopedic surgeons should consider modifiable risk factors of DVT and prophylactic medication after TKA.

Although it remains to be determined whether incidentally detected vascular pathology is significantly related to clinical outcomes and periprosthetic joint infection, understanding the prevalence of vascular pathology and associated risk factors in TKA candidates is important for successful TKA.
